# Acute physiological and perceptual responses in the FIT FIRST FOR ALL school-based physical activity program

**DOI:** 10.1038/s41598-026-55141-4

**Published:** 2026-05-29

**Authors:** Helgi Winther Olsen, Tórur Sjúrðarson, Bára Berghamar Danielsen, May-Britt Skoradal, Peter Krustrup, Magni Mohr, Malte Nejst Larsen

**Affiliations:** 1 Faculty of Education, University of the Faroe Islands, Tórshavn, Faroe Islands; 2 Center of Health Science, Faculty of Health, University of the Faroe Islands, Tórshavn, Faroe Islands; 3 Department of Sports Science and Clinical Biomechanics, SDU Sport and Health Sciences Cluster (SHSC), Faculty of Health Sciences, University of Southern Denmark, Odense, Denmark; 4 Danish Institute for Advanced Study (DIAS), University of Southern Denmark, Odense, Denmark

**Keywords:** School-based physical activity, Acute physiological and perceptual responses, Exercise intensity, Health-related fitness, Physical activity enjoyment, Lifestyle modification, Public health, Quality of life

## Abstract

This study examined acute physiological and perceptual responses to the FIT FIRST FOR ALL program, a structured school-based physical activity intervention designed to promote engagement. In a non-randomized cluster study conducted in the Faroe Islands, 179 pupils aged 7–16 participated in three weekly 40-minute sessions for 10 weeks as part of a school-wide program alongside physical education. Acute responses were assessed in a subsample (*n* = 53) using heart rate, accelerations, perceived exertion, and enjoyment, with individualized thresholds and stratification based on baseline cardiorespiratory fitness assessed using a shuttle run test. Sessions consistently elicited moderate-to-vigorous intensity, with mean heart rate ranging from 67.2 ± 1.3 to 69.9 ± 1.4% of maximal heart rate. Pupils spent 57.9 ± 3.6 to 64.9 ± 3.3% of session time at moderate-to-vigorous intensity, including up to 31.8 ± 3.0% at vigorous intensity. Despite higher physiological and perceived exertion among pupils in the low fitness group, enjoyment was comparable across fitness groups. Cardiorespiratory fitness improved among the intervention sample (*n* = 117), with a tendency for greater gains in the lowest fitness tertile, although differences were not statistically significant (*p* = 0.063, ε² = 0.024). These findings suggest that FIT FIRST FOR ALL can elicit health-promoting intensity and high enjoyment across age and fitness groups when delivered school-wide through teacher-led sessions.

## Introduction

Despite growing evidence of the benefits of physical activity for children’s physical, mental, and social health, global participation rates remain low^1–3^. Recent harmonized surveillance data confirm that these low levels have remained stable and undesirable over the past decade across most regions^4^. In response, the World Health Organization and UNESCO have called for scalable, context-specific strategies to improve physical activity levels in youth worldwide, emphasizing inclusive, developmentally appropriate, and engaging programs delivered through schools^5–7^.

Schools offer a uniquely powerful setting for such interventions, as they provide structured and equitable access to all children regardless of background, gender, or fitness level^2,3,8^. However, physical education classes often vary widely in content and intensity and are insufficient to meet recommended daily activity levels^9,10^. Pupils with lower fitness tend to be less active and less engaged in typical PE^11^, highlighting the importance of structured programs designed to support participation and perceived competence across fitness levels.

Recent meta-analyses have identified several features of school-based physical activity interventions that are most likely to improve components of health-related fitness, including cardiorespiratory fitness, as well as broader health outcomes in youth. Structured and standardized delivery, a frequency and duration typically of two to three sessions per week over at least eight to twelve weeks, moderate-to-vigorous intensity verified through objective measures, and developmentally appropriate design are consistently reported as effective^11–14^. Programs are also more effective when implemented school-wide, supported by trained staff, and designed to target specific outcomes such as cardiovascular fitness, strength, or motor competence rather than relying on general activity exposure^5^. High-intensity interval formats appear particularly beneficial for cardiorespiratory fitness and cardiometabolic health^15,16^, while program effectiveness also depends on fidelity and delivery quality^17,18^. Comprehensive reviews confirm effectiveness when interventions are delivered with structure and inclusivity^19^, although effects may be smaller or absent in adolescents^20^ and may differ by gender^2^. Importantly, well-designed interventions should engage not only the already active but also those who are least fit or least confident, who often benefit the most when included but are frequently underserved^21^.

The FIT FIRST FOR ALL program investigated in the present study was developed to complement traditional physical education by providing structured, high-intensity activity sessions that are ready to implement^22^. Unlike general PE or movement initiatives that rely on open-ended or teacher-driven design, FIT FIRST FOR ALL offers pre-planned, sport-themed games designed to elicit moderate-to-vigorous physical activity. The program is built for inclusivity and progression, with age-specific formats FIT FIRST 10, 20, and Teen (FF10, FF20, FFT) targeting children aged 7 to 16. Its evolving, open-source session library and ready-to-use structure make the program freely available for all schools, while teacher training is offered to ensure that trained educators can deliver it consistently across diverse settings. This structure directly reflects recommendations for well-designed school-based programs designed to improve health-related fitness, emphasizing structured delivery, targeted intensity and volume, and additional sessions alongside standard PE^13,14,23^. It also embodies the principles set out in the UNESCO Quality Physical Education rationale, the Copenhagen Consensus Conference, and Danish public health priorities, all of which highlight the need for equitable access to structured, health-enhancing physical activity during the school day^6,7,23,24^.

While a recent study found no health-related fitness effects of the FIT FIRST Teen program implemented over a 10-week period in Danish schools for pupils aged 12 to 15 years^25^, a study investigating a 10-week school-wide implementation of all three FIT FIRST FOR ALL formats in a Faroese school for pupils aged 7 to 16 reported significant improvements. Cardiorespiratory fitness increased by 31% and agility by 2%, while muscle mass rose by 7% and body fat decreased by 9%, expressed as relative changes from baseline to post-intervention^26^. Physical well-being also improved by 7%, with no changes in other health-related quality of life domains^27^. These findings highlight the potential of the FIT FIRST FOR ALL program when implemented school-wide, although outcomes may differ across studies and delivery settings. To advance understanding, it is necessary to look beyond pre–post changes and investigate whether the sessions achieved the intended intensity and how pupils experienced them. Examining acute physiological and perceptual responses provides insight into the mechanisms that may explain how such programs influence fitness and well-being.

The present study examines pupils’ acute physiological and perceptual responses during FIT FIRST FOR ALL sessions, with a focus on intensity and whether this varies by age and baseline fitness. Acute responses provide a window into how children are physically active and engaged during sessions, particularly those most at risk of being left behind. Perceived exertion and enjoyment are central not only for immediate engagement but also for building fitness, motivation, and lasting activity habits^8,28^. While some studies suggest that children may not enjoy high-intensity activity^29^, enjoyment is likely shaped by inclusion, agency, and a sense of competence^28,30,31^, consistent with the motivational principles outlined in self-determination theory^32^. These perspectives highlight equity as a core concern, since pupils with lower fitness or confidence often face the greatest barriers to participation^33,34^. Engagement can be understood as multidimensional, encompassing behavioral, emotional, cognitive, and agentic elements, making acute responses a useful lens for evaluating whether a school-based program supports all pupils consistently^35^.

This study contributes to a broader understanding of the physical targets school-based programs should aim to achieve, while also offering insight into how the FIT FIRST FOR ALL program is perceived by pupils. The primary objective is to evaluate acute physiological and perceptual responses across age groups and baseline fitness tertiles within the three FIT FIRST programs (FF10, FF20, FFT) delivered school-wide. The secondary objective is to examine whether the program elicits different acute responses and cardiorespiratory fitness changes according to baseline fitness. We hypothesize that FIT FIRST sessions will elicit health-promoting intensity while maintaining high enjoyment across age and fitness groups. We further expect that the program will elicit relative physiological and perceived exertion among pupils with lower baseline fitness comparable to that of their peers.

## Methods

### Study design

The present study investigates acute physiological and perceptual responses assessed during FIT FIRST FOR ALL physical activity sessions delivered as part of a 10-week school-based intervention. The intervention was implemented within a non-randomized cluster design^36^, in which two comparable Faroese schools served as allocation units based on pre-existing implementation decisions and were matched on size and demographic profile. One school implemented the FIT FIRST FOR ALL program alongside its standard physical education curriculum, while the other continued standard physical education and served as a passive control during the intervention period. While overall intervention effects were evaluated through comparison between the two schools, the acute responses were investigated in the intervention school.

To evaluate these acute responses, 12 sessions were observed across three age groups corresponding to the age-specific FIT FIRST program formats FF10, FF20, and FFT. One class per age group was randomly selected, and sessions in weeks 6, 7, 9, and 10 were chosen within the constraints of maintaining normal school operations while ensuring representation across age groups and capturing a broad range of session types.

In addition to the acute response analyses, health-related fitness outcomes were evaluated in the full intervention sample as part of the broader study. Cardiorespiratory fitness was assessed using the Yo-Yo Intermittent Recovery Level 1 Children’s shuttle run test^37^, which was also used to define baseline fitness tertiles for stratified analyses. This shuttle run test formed part of a standardized test battery that further included the Arrowhead Agility test^38^, the Standing Long Jump^39^, the Stork Stand balance test^40^, resting blood pressure^41^, body composition measured by bioimpedance^42^, and the KIDSCREEN-52 questionnaire assessing health-related quality of life^43^. Pre- and post-intervention changes were calculated relative to baseline values. Detailed procedures and overall intervention effects have been reported elsewhere^26,27^.

### Participants

A total of 179 pupils aged 7 to 16 in grades 1 to 9 were enrolled in the intervention school. An additional 181 pupils from a comparable control school completed the pre- and post-tests but did not participate in FIT FIRST sessions and were therefore not included in the present acute response analyses. The intervention school follows the Faroese three-level model. Level I includes grades 1 to 3 (approximately 7 to 9 years), Level II includes grades 4 to 6 (10 to 12 years), and Level III includes grades 7 to 9 (13 to 16 years).

**Fig. 1:**
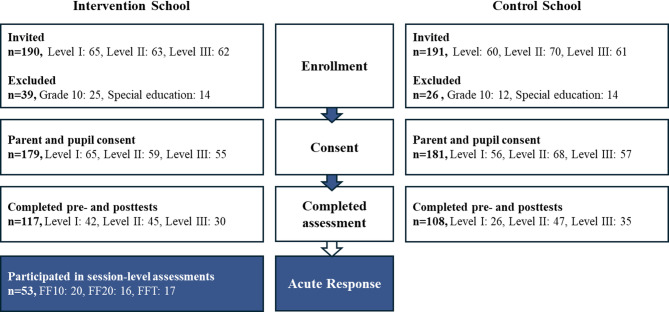
Flow of participants through enrollment, consent, assessment, and inclusion in acute and stratified fitness analyses. Pupils from the intervention school (left) and control school (right) are shown. Acute response analyses were based on a subsample of 53 pupils, while stratified fitness change analyses included all intervention pupils with valid pre- and posttest shuttle run data (*n* = 117). Control school data were not included in the present analyses.

For the primary objective of evaluating acute responses, one full class was randomly selected from each age group, yielding a subsample of 53 pupils: Level I (*n* = 20, 8.6 ± 0.3 years), Level II (*n* = 16, 10.7 ± 0.3 years), and Level III (*n* = 17, 13.5 ± 0.3 years). All pupils in the selected classes participated in the 12 observed sessions analyzed for acute responses. Descriptive characteristics of this subsample are presented in Table 1, and the overall flow of participants through enrollment, testing, and inclusion in acute and stratified analyses is shown in Fig. 1.

**Table 1:** Participant characteristics of the acute response subsample (*n* = 53) by FIT FIRST programs (FF10, FF20, FFT).

Program	*n*	Age	Gender	Height	Weight	BMI
		yr	m/f (%)	cm	kg	kg/m^2^
FF10	20	8.6 ± 0.3	75/25	134 ± 6	34.1 ± 8.4	18.9 ± 3.5
FF20	16	10.7 ± 0.3	63/37	147 ± 7	45.0 ± 13.7	20.5 ± 4.7
FFT	17	13.5 ± 0.3	53/47	165 ± 8	58.0 ± 12.1	21.2 ± 3.2

Values are presented as mean ± SD, except for gender, which is reported as percentages. BMI = body mass index.

For the secondary objective of investigating the role of baseline fitness, analyses drew on the full intervention population (*N* = 179) for pre- to post-test changes in fitness, while stratified acute response analyses were conducted within the subsample (*n* = 53).

### Intervention delivery

During the 10-week intervention period, pupils at the intervention school received three weekly 40-minute FIT FIRST sessions in addition to regular physical education. Sessions were delivered in school gym facilities (approximately 20 × 10 m). Each week featured a different sport-themed activity, pre-structured within the FIT FIRST library and randomized by the research team to ensure thematic variety. The program was delivered in three age-adapted formats that corresponded with the Faroese school levels. FF10 was used in Level I covering grades 1 to 3, FF20 was used in Level II covering grades 4 to 6, and FFT was used in Level III covering grades 7 to 9. Before implementation, trained PE teachers completed a FIT FIRST educational course led by the research team to ensure consistency in delivery and adherence to the program format.

### Evaluation framework

This analysis was guided by the internal–external training load framework^44^, which distinguishes between the external demands of activity and the individual’s internal response. To capture these dimensions, we evaluated four session parameters, with mean heart rate representing internal load, acceleration rate representing external load, perceived exertion (RPE) representing subjective load, and enjoyment representing affective response, assessed using the How Happy Scale (HHS). The intensity-based variables heart rate, acceleration, and RPE were analyzed using individualized thresholds and categorized within a unified three-zone model (light, moderate, vigorous), to assess alignment with physical activity guidelines^45^, as presented in Table 2. Enjoyment (HHS) was not intensity-based and was evaluated separately using its four-category scale.

**Table 2:** Intensity-based session parameters categorized within the unified three-zone model.

	Light	Moderate	Vigorous
Heart rate	< 64%	64–76%	> 76%
Rating of Perceived Exertion	0–4 RPE	5–6 RPE	7–10 RPE
Acceleration	20–44%	45–84%	> 85%

Heart rate zones based on the American College of Sports Medicine and cut offs for rating of perceived exertion (RPE) were adapted from Coates et al., 2023^46^. Acceleration thresholds were adapted from Johnston et al., 2014^49^. All thresholds are expressed relative to each participant’s individual maximum values.

### Internal load (Heart rate)

Internal load was assessed continuously during each observed FIT FIRST session using the Polar Team Pro system (Polar Electro Oy, Kempele, Finland), which recorded heart rate data at 10 Hz. Trained research assistants ensured correct placement of the chest straps, positioning each sensor directly on the skin below the sternum and adjusting it for comfort and stability. Female researchers assisted girls and male researchers assisted boys to ensure comfort and appropriateness during sensor placement. Heart rate values were expressed relative to each participant’s highest recorded heart rate observed during either the shuttle run test or FIT FIRST sessions. Intensity distributions were classified using a unified three-zone model adapted from ACSM guidelines^46^ and Coates et al. (2023)^45^, defining light intensity as < 64%HR_max_, moderate as 64–76%HR_max_, and vigorous as > 76%HR_max_.

### External load (Acceleration)

External load was assessed continuously during each observed session using the Polar Team Pro system’s inbuilt inertial movement unit (Polar Electro Oy, Kempele, Finland) to estimate translational acceleration in the horizontal plane, expressed in meters per second squared (m·s^− 2^). This chest-worn setup and the method for deriving accelerations have been validated for use in sports-based tasks^47^. Acceleration rate was defined as the number of accelerations per minute during each session. Intensity distributions were classified using individualized thresholds derived from peak accelerations observed during the pre-test shuttle run, providing a relative reference for acceleration demands during sessions. These thresholds were adapted from a three-zone framework originally using sprint-derived acceleration values calculated over fixed distances^48^, defining light intensity as 20–44% of individual peak acceleration, moderate as 45–84%, and vigorous as > 85%. Although this approach has not been formally validated for m·s^− 2^-based acceleration in children, it provided a practical framework to classify external load using individualized thresholds.

### Perceptual and affective measures

Perceived exertion and enjoyment during FIT FIRST sessions were assessed with the Children’s OMNI Scale of Perceived Exertion (RPE) and the How(e) Happy Scale (HHS). The instruments were administered together on a paper questionnaire printed double-sided in color and completed immediately after each session in the gym setting, which typically required one to two minutes. Instructions were given to the group by a researcher or trained teacher, who supervised completion and provided neutral clarification when needed. Both instruments were originally published in English and were translated into Faroese using two independent forward translations, one back-translation, and pre-testing with children aged 8 to 12 to ensure clarity and cultural relevance^49^.

#### Perceived exertion (RPE)

The OMNI scale is a validated 10-point visual analog scale with pictorial anchors representing increasing levels of physical strain^50,51^. The stepping version was selected for its relevance to the varied movements in FIT FIRST sessions. Prior evidence indicates that gender-specific pictograms on the OMNI scale do not affect validity^52^, and a single pictogram version was randomly selected and used for all participants. For analysis, RPE responses were grouped into light (0–4), moderate (5–6), and vigorous (7–10), consistent with the unified three-zone intensity model applied also to heart rate and acceleration^45^.

#### Enjoyment (HHS)

Enjoyment, as an affective dimension of physical activity engagement, was assessed as a state response using the How(e) Happy Scale (HHS), a validated four-point facial-expression scale that captures four domains representing attraction, preference, social experience, and environmental perception. The scale has been validated for use in children aged 7 to 13 in sport and play contexts. Enjoyment was calculated as a composite score by averaging the four domain responses, and domain score distribution was presented descriptively^29^.

## Analyses

### Data processing

Heart rate and acceleration data were exported from the Polar Team Pro platform and processed using MATLAB (R2024a, The MathWorks Inc., Natick, MA, USA). For each pupil, individualized thresholds were established based on peak acceleration values from pre-test shuttle runs and the highest recorded heart rate observed during either the shuttle run test or FIT FIRST sessions. These thresholds were used to calculate session metrics including mean heart rate, peak heart rate, acceleration rate, and time spent in light, moderate, and vigorous intensity zones, classified according to the unified three-zone model^45^, with individualized thresholds ensuring that zones reflected relative effort for each pupil. All processed metrics were then prepared for statistical analysis in R (R Core Team, v4.3.1).

### Acute response analysis

To address the first objective, differences in heart rate, acceleration, perceived exertion, and enjoyment were examined across sessions and across the three FIT FIRST programs (FF10, FF20, FFT). Within each program, linear mixed-effects models were fitted with session (categorical) as a fixed effect and participant ID as a random effect with random intercepts, allowing each participant to contribute repeated measures with their own baseline level. This structure also accounted for unbalanced participation due to missing sessions^53,54^. No imputation of missing session data was performed. Model assumptions were evaluated using residual diagnostics. Sidak-adjusted pairwise comparisons of estimated marginal means were used to identify differences between sessions. Differences between FIT FIRST programs were evaluated with participant means across sessions analyzed by one-way ANOVA with Sidak-adjusted pairwise comparisons. Intensity distributions were analyzed using a unified three-zone model (light, moderate, vigorous)^45^ applied to heart rate, acceleration, and perceived exertion, based on individualized thresholds defined during data processing. Enjoyment was analyzed separately as a non-intensity-based measure. Analyses were conducted in R (R Core Team, v4.3.1) using the *lmerTest*, *emmeans*, and *rstatix* packages^55–57^.

### Fitness-stratified analyses

To address the secondary objective, participants were stratified into age group–specific tertiles of baseline fitness, derived from shuttle run performance (low, moderate, high). Tertiles were treated as categorical groups, and differences in relative improvement (pre–post shuttle run distance) and acute responses (heart rate, acceleration, perceived exertion, enjoyment) were assessed using Kruskal–Wallis rank sum tests with Bonferroni-adjusted Dunn post hoc comparisons^58,59^, which were chosen to accommodate non-normal distributions and unequal group sizes. Complementary analyses treated baseline fitness as a continuous predictor, with associations to acute responses examined using Spearman’s rank correlation coefficients^60^. Relationships were visualized with scatterplots and linear smoothing. All pupils with valid pre- and post-test data (*n* = 117 of 179) were included in analyses of fitness change, while the acute response subsample (*n* = 53) was analyzed separately for outcomes across sessions and programs. All analyses were performed in R (R Core Team, v4.3.1) using the *rstatix* and *dunn.test* packages^61,62^.

### Ethics approval

This study was approved by the Research Ethics Council of the Faroe Islands (No. 2023-08). All procedures were conducted in accordance with relevant guidelines and the Declaration of Helsinki. Written informed consent was obtained from legal guardians, and pupils were informed that participation in the research was voluntary.

## Results

All outcomes are reported as estimated marginal means with standard errors unless otherwise noted. The results are presented by outcome domain, beginning with acute responses across sessions and programs, followed by fitness-stratified analyses of improvements in shuttle run performance and associations between baseline fitness and acute responses.

### Acute responses across programs

#### Heart rate

**Fig. 2:**
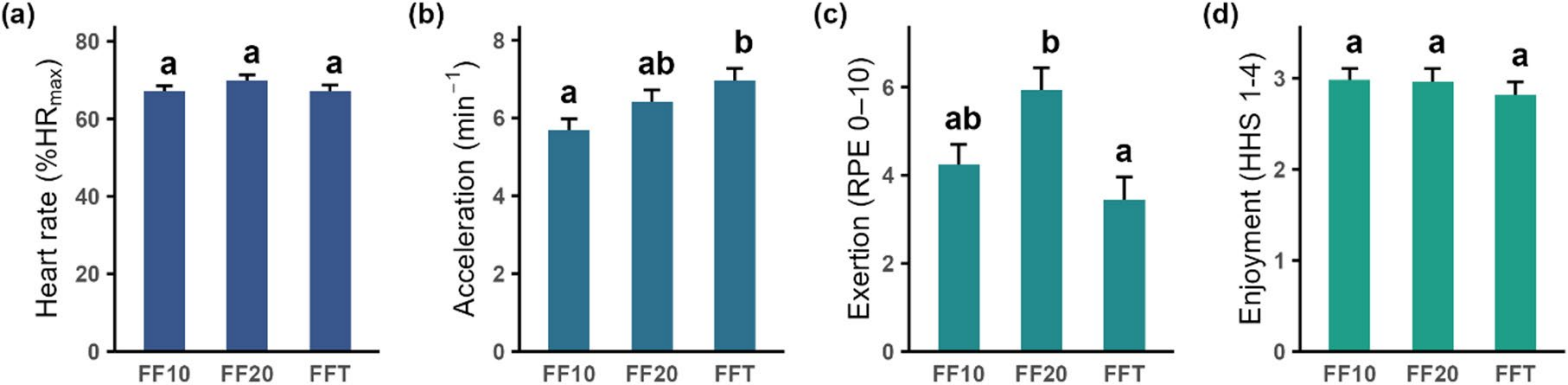
Physiological and perceptual acute responses across FIT FIRST programs (FF10, FF20, FFT). Bars show group means ± SE for (**a**) mean heart rate (%HR_max_, relative to maximum heart rate), (**b**) mean acceleration rate (min⁻¹), (**c**) perceived exertion (RPE, 0–10), and (**d**) enjoyment (How Happy Scale, HHS, 1–4). Different superscript letters indicate significant differences between programs (*p* < 0.05).

With heart rate expressed relative to individual maximum heart rate, mean values across programs were 67.2 ± 1.3% in FF10, 69.9 ± 1.4% in FF20, and 67.3 ± 1.4% in FFT, with no significant differences (*p* = 0.12). Peak values showed the same pattern, with 89.7 ± 1.2% in FF10, 91.1 ± 1.3% in FF20 and 88.9 ± 1.3% in FFT (*p* = 0.26). Pupils spent between 57.9 ± 3.6% and 64.9 ± 3.3% of time at moderate-to-vigorous intensity and between 21.5 ± 2.5% and 31.8 ± 3.0% at vigorous intensity, without significant differences across programs (Fig. 2A; Table 3).

**Table 3:** Physiological, perceptual, and enjoyment responses during FIT FIRST sessions across programs (FF10, FF20, FFT).

		FF10	FF20	FFT
**Heart rate**		*n* = 19	*n* = 16	*n* = 16
Mean heart rate	%HR_max_	67.2 ± 1.3 (a)	69.9 ± 1.4 (a)	67.3 ± 1.4 (a)
Peak heart rate	%HR_max_	89.7 ± 1.2 (a)	91.1 ± 1.3 (a)	88.9 ± 1.3 (a)
Moderate-to-vigorous	%	57.9 ± 3.6 (a)	64.9 ± 3.3 (a)	57.2 ± 4.9 (a)
Vigorous	%	21.5 ± 2.5 (a)	31.8 ± 3.0 (a)	27.7 ± 4.3 (a)
**Acceleration**		*n* = 19	*n* = 16	*n* = 16
Mean acceleration	min^− 1^	5.7 ± 0.3 (a)	6.4 ± 0.3 (ab)	7.0 ± 0.3 (b)
Moderate-to-vigorous	%	21.1 ± 3.6 (a)	23.7 ± 2.4 (a)	22.8 ± 2.6 (a)
Vigorous	%	2.4 ± 0.5 (a)	2.8 ± 0.7 (a)	1.9 ± 0.4 (a)
**Exertion**		*n* = 20	*n* = 16	*n* = 16
RPE	0–10	4.2 ± 0.5 (ab)	5.9 ± 0.5 (b)	3.4 ± 0.5 (a)
Moderate-to-vigorous	%	36.7 ± 8.4 (a)	71.9 ± 11.2 (a)	42.2 ± 9.1 (a)
Vigorous	%	23.8 ± 7.0 (a)	42.2 ± 9.1 (b)	0.0 ± 0.0 (a)
**Enjoyment**		*n* = 20	*n* = 16	*n* = 16
HHS	1–4	3.0 ± 0.1 (a)	3.0 ± 0.1 (a)	2.8 ± 0.1 (a)
Attraction	1–4	2.9 ± 0.2 (a)	2.6 ± 0.2 (a)	2.7 ± 0.2 (a)
Environment	1–4	2.9 ± 0.2 (a)	2.6 ± 0.2 (a)	2.7 ± 0.2 (a)
Preference	1–4	3.1 ± 0.1 (a)	3.4 ± 0.2 (a)	2.8 ± 0.2 (a)
Social	1–4	3.1 ± 0.1 (a)	3.2 ± 0.2 (a)	3.1 ± 0.2 (a)

Values are group means ± SE for mean and peak heart rate (both relative to HR_max_), mean acceleration rate (min⁻¹), perceived exertion (rating of perceived exertion, RPE, 0–10), and enjoyment (How Happy Scale, HHS, 1–4). Zone distributions are presented for heart rate, acceleration, and exertion, where moderate-to-vigorous represents the combined time in moderate and vigorous intensity and vigorous represents vigorous intensity only. Enjoyment is further separated into the four components of the How Happy Scale (Attraction, Environment, Preference, Social). Different letters indicate significant differences between programs (*p* < 0.05).

**Fig. 3:**
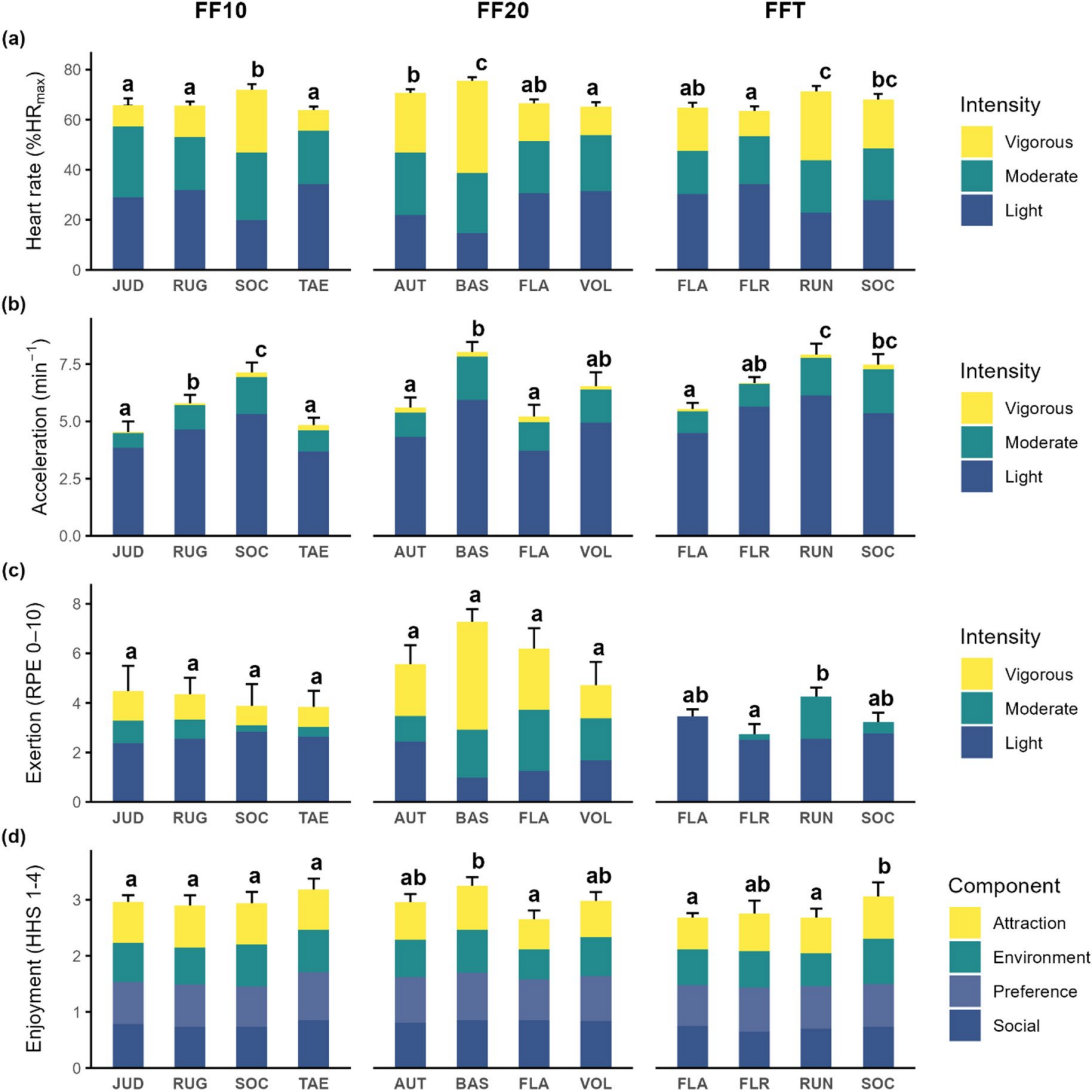
Physiological and perceptual acute responses across sessions within each FIT FIRST program (FF10, FF20, FFT). Bars show group means ± SE for (**a**) mean heart rate (%HR_max_, relative to maximum heart rate), (**b**) mean acceleration rate (min⁻¹), (**c**) perceived exertion (rating of perceived exertion, RPE, 0–10), and (d) enjoyment (How Happy Scale, HHS, 1–4). Zone distributions (light, moderate, vigorous) and enjoyment component distributions are shown within the bars. Session labels indicate FIT FIRST activity themes: JUD = Judo, RUG = Rugby, SOC = Soccer, TAE = Taekwondo, AUT = Automobile, BAS = Basketball, FLA = Flag Football, VOL = Volleyball, FLR = Floorball, RUN = Running. Different superscript letters indicate significant differences between sessions within each program (*p* < 0.05).

Within programs, mean heart rate in FF10 ranged from 63.9 ± 1.4% in taekwondo to 72.8 ± 1.4% in soccer (*p* < 0.001). In FF20, values ranged from 65.5 ± 1.4% in volleyball and the highest was 75.5 ± 1.4% in basketball (*p* < 0.001). In FFT, values ranged from 63.2 ± 2.2% in floorball to 71.4 ± 2.1% in running (*p* < 0.001). Peak heart rate followed similar patterns. In FF10, values ranged from 85.9 ± 1.8% in taekwondo to 92.4 ± 2.0% in soccer but these differences were not significant (*p* = 0.077). In FF20, values ranged from 86.1 ± 1.6% in volleyball to 94.5 ± 1.6% in basketball (*p* < 0.001). In FFT, values ranged from 83.8 ± 2.0% in floorball to 92.1 ± 1.9% in soccer (*p* = 0.006). Session results and intensity distributions are shown in Fig. 3A.

#### Acceleration

Mean acceleration rate across programs was 5.7 ± 0.3 min^− 1^ in FF10, 6.4 ± 0.3 min^− 1^ in FF20, and 7.0 ± 0.3 min^− 1^ in FFT, with FFT significantly higher than FF10 (*p* = 0.041). Pupils spent 21.1 ± 3.6% to 23.7 ± 2.4% of time at moderate-to-vigorous acceleration and 1.9 ± 0.4% to 2.8 ± 0.7% at vigorous acceleration, with no significant differences across programs (Fig. 2B; Table 3).

Within programs, acceleration rates ranged from 4.6 ± 0.4 min^− 1^ in judo to 7.2 ± 0.3 min^− 1^ in soccer in FF10 (*p* = 0.003), from 5.2 ± 0.5 min^− 1^ in flag football to 8.0 ± 0.5 min^− 1^ in basketball in FF20 (*p* < 0.001), and from 5.3 ± 0.5 min^− 1^ in automobile to 8.0 ± 0.4 min^− 1^ in running in FFT (*p* < 0.001). Session results and intensity distributions are shown in Fig. 3B.

#### Perceived exertion

Mean RPE across programs was 4.2 ± 0.5 in FF10, 5.9 ± 0.5 in FF20, and 3.4 ± 0.5 in FFT, with FF20 significantly higher than FFT (*p* = 0.007) and FF10 not significantly different from either. Pupils rated between 36.7 ± 8.4% and 71.9 ± 11.2% of time at moderate-to-vigorous exertion and between 23.8 ± 7.0% and 42.2 ± 9.1% at vigorous exertion in FF10 and FF20, with FF20 significantly higher than FF10 (*p* = 0.041) and FFT (*p* < 0.001), and pupils in FFT rated sessions as vigorous (Fig. 2C; Table 3).

Within programs, RPE in FF10 ranged from 3.8 ± 0.7 in taekwondo to 4.8 ± 0.7 in judo, with no significant differences. In FF20, values ranged from 4.9 ± 0.8 in volleyball to 7.0 ± 0.8 in basketball, without significant differences. In FFT, values ranged from 2.8 ± 0.4 in floorball to 4.3 ± 0.4 in running, with running significantly higher than floorball (*p* = 0.043). Session results and intensity distributions are shown in Fig. 3C.

#### Enjoyment

Composite enjoyment scores were consistent across programs, with values of 3.0 ± 0.1 in FF10, 3.0 ± 0.1 in FF20, and 2.8 ± 0.1 in FFT, with no significant differences (*p* = 0.34). The four domain scores (attraction, preference, social experience, environmental perception) were equal across categories, supporting the use of composite scores as the primary outcome (Fig. 2D; Table 3).

Within programs, composite scores in FF10 ranged from 2.9 ± 0.2 in judo to 3.2 ± 0.2 in taekwondo, with no significant differences. In FF20, values ranged from 2.7 ± 0.2 in flag football to 3.3 ± 0.2 in basketball, with basketball significantly higher than flag football (*p* = 0.044). In FFT, values ranged from 2.6 ± 0.2 in flag football to 3.1 ± 0.2 in soccer, with soccer significantly higher than flag football (*p* = 0.004). Session results are shown in Fig. 3D.

### Responses by baseline fitness

#### Fitness improvements

**Fig. 4:**
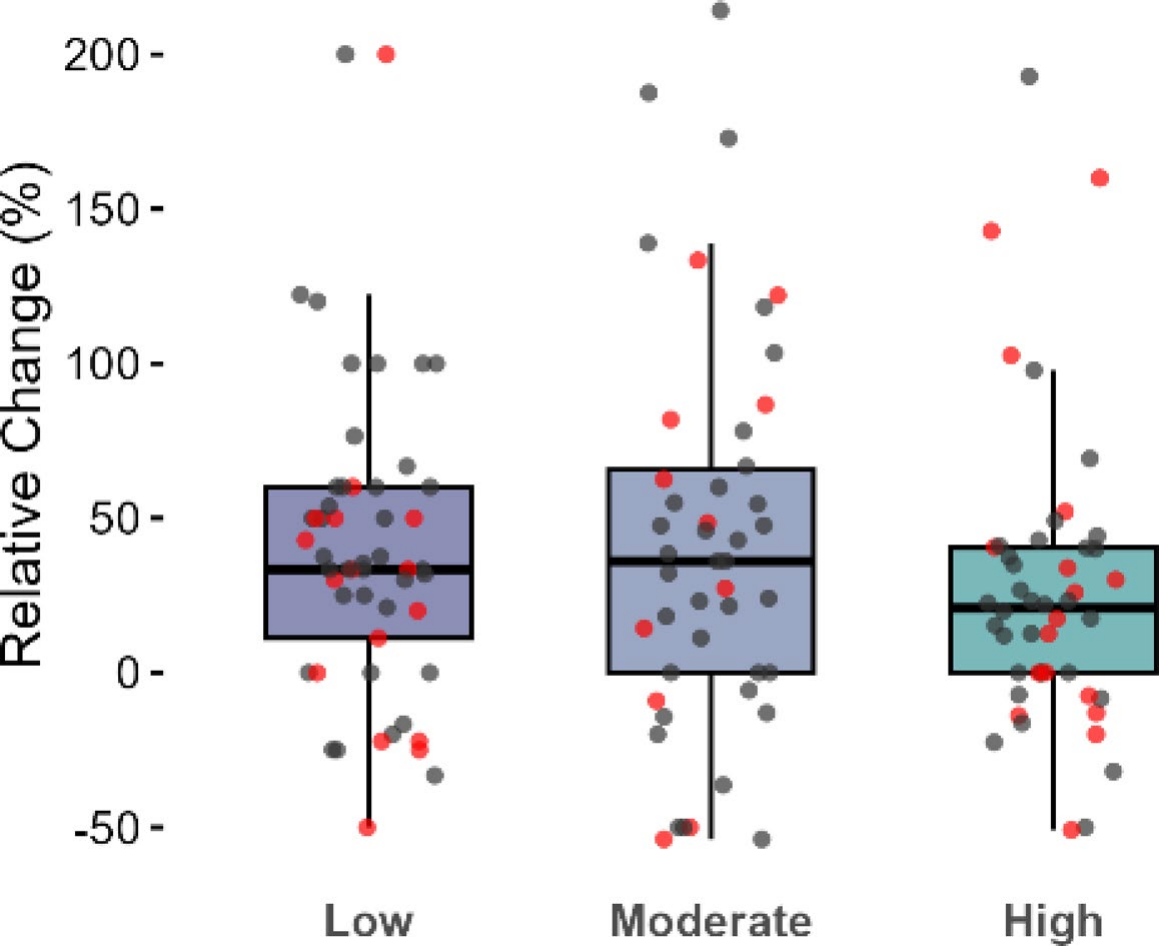
Relative improvements in fitness across baseline fitness tertiles. Boxplots show relative change (%) in distance covered in the shuttle run test from pre- to post-intervention for low, moderate, and high fitness groups, based on 117 of the 179 intervention pupils who had valid pre- and post-test data. Red points denote the acute response subsample, of whom 44 of 53 pupils had valid pre- and post-test data (FF10 = 17, FF20 = 15, FFT = 12). Differences between groups, including all intervention pupils, were not statistically significant (*p* = 0.063).

Pupils were grouped into fitness tertiles within each age group based on pre-intervention shuttle run performance. At baseline, values in the intervention group ranged from 149 ± 10 m in the low fitness group to 742 ± 78 m in the high fitness group in FF10, from 204 ± 12 m to 1712 ± 86 m in FF20, and from 432 ± 35 m to 1943 ± 140 m in FFT. To account for the large spread in baseline performance across programs and fitness groups, improvements are reported as relative values. A total of 117 of the 179 intervention pupils had valid pre- and post-test data and were included in this analysis. Median values and interquartile ranges are reported due to the non-normal distribution of change scores. Median relative improvement in cardiorespiratory fitness across age groups was 36.4% (IQR 17.8–60.0) in the low fitness group, 36.1% (IQR 0.0–65.6) in the moderate group, and 20.9% (IQR 0.0–40.4) in the high fitness group. Although the pattern suggested greater gains among the least fit, the difference was not statistically significant (*p* = 0.063, ε² = 0.024; Fig. 4).

#### Acute responses

**Fig. 5:**
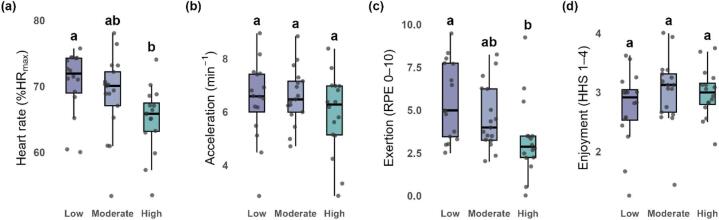
Associations between baseline fitness and acute responses. Scatterplots show individual pupil values for (**a**) mean heart rate (%HR_max_, relative to maximum heart rate), (**b**) mean acceleration rate (min⁻¹), (**c**) perceived exertion (rating of perceived exertion, RPE, 0–10), and (**d**) enjoyment (How Happy Scale, HHS, 1–4). Baseline fitness is expressed as percentage of maximum shuttle run test distance. Lines represent linear fits with 95% confidence intervals.

**Fig. 6:**
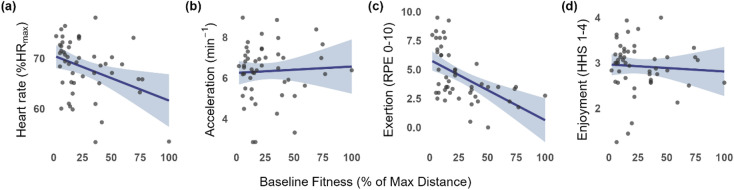
Acute responses across baseline fitness tertiles. Boxplots show distributions for low, moderate, and high fitness groups in (**a**) mean heart rate (%HR_max_, relative to maximum heart rate), (**b**) mean acceleration rate (min⁻¹), (**c**) perceived exertion (rating of perceived exertion, RPE, 0–10), and (**d**) enjoyment (How Happy Scale, HHS, 1–4). Different superscript letters indicate significant differences between groups (*p* < 0.05).

For heart rate, pupils in the low fitness group reached mean values of 69.2 ± 1.5% compared with 64.5 ± 1.4% in the high fitness group (*p* = 0.003; Fig. 5A). Peak heart rate showed the same pattern (*p* = 0.017; Fig. 5A). Mean heart rate was inversely correlated with baseline fitness (*r* = − 0.28, *p* = 0.045; Fig. 6A).

For acceleration, rates were 6.4 ± 0.5 min^− 1^ in the low fitness group and 6.7 ± 0.6 min^− 1^ in the high fitness group, with no significant differences (*p* = 0.476; Fig. 5B). No correlation was observed with baseline fitness (*r* = − 0.09, *p* = 0.52; Fig. 6B).

For perceived exertion, values were 5.3 ± 0.4 in the low fitness group and 3.6 ± 0.4 in the high fitness group (*p* = 0.009). The moderate fitness group reported 4.6 ± 0.4, which did not differ significantly from either the low (*p* = 0.24) or high group (*p* = 0.13; Fig. 5C). RPE was negatively correlated with baseline fitness (*r* = − 0.35, *p* = 0.018; Fig. 6C).

For enjoyment, scores ranged from 2.8 to 3.0 on the 1–4 scale, with no significant differences between groups (*p* = 0.312; Fig. 5D). No correlation was observed with baseline fitness (*p* = 0.73; Fig. 6D).

## Discussion

This study evaluated acute physiological and perceptual responses during FIT FIRST FOR ALL sessions in a school-wide, teacher-led setting. The discussion examines how patterns across program formats and baseline fitness inform interpretation of session intensity, enjoyment, and positioning within the broader literature on school-based physical activity.

### Acute response across programs

FIT FIRST sessions consistently elicited moderate-to-vigorous intensity, with mean heart rates between 67.2 and 69.9%HR_max_ and high enjoyment scores across age groups. These responses fall within the range associated with improvements in cardiorespiratory fitness^12,13^ and are consistent with syntheses showing that high-intensity interval formats can improve fitness and cardiometabolic health in school-aged populations^15,16^. The program’s structure, consisting of three weekly 40-minute sessions over 10 weeks, was sufficient to deliver health-promoting activity while maintaining a positive affective experience, suggesting a balance between intensity and enjoyment within the school setting. Importantly, these responses were observed across the cohort.

Although this study did not include a direct comparison with regular PE, previous literature indicates that PE lessons often fall below recommended moderate-to-vigorous physical activity (MVPA) levels. Meta-analyses report that elementary pupils spend on average 44.8% of lesson time in MVPA^9^, while secondary pupils average just 40.5%^64^, both below the 50% guideline recommended by international authorities^63^. In the present study, pupils accumulated 57 to 65% of session time at moderate-to-vigorous intensity, corresponding to approximately 23 to 26 min per 40-minute session depending on age group. These values exceed typical PE averages reported in the literature and demonstrate that structured sessions can reach guideline-aligned intensity within a school timetable. Ball-based session themes such as soccer and basketball were associated with higher internal and external loads and high enjoyment, while not being perceived as more exertive, consistent with previous findings from small-sided game formats in school settings^64–67^. These findings align with more recent syntheses indicating that game-based high-intensity formats can effectively combine cardiovascular stimulus and enjoyment in youth^15^. However, all session themes followed the same inclusive structure, and enjoyment remained high even in more demanding sessions. These findings are consistent with theories linking enjoyment to future participation^8,28^ and suggest that structured session formats can support both effort and positive experience in school-based settings.

Importantly, the goal of school-based physical activity extends beyond short-term achievement of MVPA targets. Maximizing activity through continuous drills may compromise motivation and engagement^68^. FIT FIRST FOR ALL was designed to combine structured intensity with inclusive and meaningful session experiences. This approach aligns with international recommendations emphasizing inclusion, enjoyment, and personal development over performance^7^ and is consistent with self-determination theory, which identifies autonomy, competence, and relatedness as essential for sustained motivation^32^. Evidence from school PE contexts indicates that autonomy-supportive strategies are associated with higher engagement and well-being^31,69^, and longitudinal research suggests that motivational mechanisms rooted in SDT predict changes in daily activity among at-risk adolescents^69,70^. Approaches that prioritize engagement and positive experience alongside intensity may strengthen the longer-term sustainability of structured school-based programs^2,65,71^.

### Acute responses by baseline fitness

A secondary objective of the study was to examine whether acute physiological and perceptual responses differed according to baseline fitness, and specifically whether the program engaged pupils with lower baseline fitness comparably to their peers. These pupils consistently reached higher mean and peak heart rates and reported greater exertion than fitter peers, yet showed no reduction in enjoyment. External load did not differ across fitness groups, indicating that similar movement demands were imposed despite differences in internal and perceived responses. This suggests successful inclusion and activation under the session conditions studied. Given the well-documented difficulty of reaching low-fitness children in traditional settings^21,72^, this pattern is noteworthy.

Although greater relative fitness gains among low-fit pupils are expected because improvements are typically larger when baseline levels are low, there was a tendency for greater gains in the lowest fitness tertile, although differences between fitness groups were not statistically significant and were small in magnitude. Across the intervention sample, cardiorespiratory fitness improved while enjoyment remained high, suggesting that the sessions were engaging enough to sustain participation across the intervention period. Importantly, enjoyment remained high even when internal and perceived loads were elevated, suggesting the program successfully balanced challenge with positive experience^28,29^. This reflects engagement across both behavioral and emotional dimensions, consistent with broader perspectives that define engagement in PE as multidimensional^35^.

This also opens a broader reflection on why the program may have functioned as observed. One factor may be that pupils with low fitness are less visible in PE, as their effort is not always recognized or rewarded in traditional performance-based formats^73^. While we did not examine this directly, the consistently high enjoyment scores in our study suggest that pupils, including those with low fitness, felt included during sessions. The simple, game-based design and consistent structure align with broader arguments that contextual and pedagogical features are central to inclusive physical education and Quality Physical Education frameworks^7,74,75^. One possible explanation is that these sessions created an environment where effort was internalized and normalized rather than singled out or judged, illustrating how principles aligned with self-determination theory may have supported pupils in sustaining effort while maintaining enjoyment^32,69^.

When combined with a motivated teaching staff and a supportive school environment, these factors may help explain why the program appeared able to engage pupils across fitness levels. Teacher training, confidence, and motivation are critical for program quality and sustainability^76^, and supportive school settings with clear implementation practices are essential to ensure that physical activity programs translate into meaningful outcomes^77^.

### Translational relevance of the program

Evidence from related contexts shows that outcomes cannot be assumed to generalize without supportive implementation conditions. A recent Danish study found no effect of the FIT FIRST Teen program on fitness when implemented over ten weeks^25^, and the FIT FIRST 10 dose-response trial highlighted barriers such as timetable pressure, limited facilities, and modest teacher motivation, especially when delivery was led by teachers without a formal PE education^78^. The Generation Healthy Kids study, which included FIT FIRST as a core component of a broader health intervention, further demonstrated that frontline workers’ capability, opportunity, and motivation were shaped by organizational context, with leadership support emerging as decisive for successful delivery^79^.

These findings align with broader implementation research showing that program effectiveness depends on structured delivery, sufficient intensity, and progression^21,80^. Successful implementation also appears more likely in environments characterized by educated PE teachers, leadership engagement, and whole-school alignment^74,81^. In the present study, acute responses indicated that teacher-led sessions consistently reached moderate-to-vigorous intensity while maintaining high enjoyment across age and fitness groups. This occurred within a school-wide setting delivered by educated PE teachers trained in the program and supported by school leadership, which may help explain why the program reached its intended physiological and perceptual targets^19,76^. Accordingly, the transferability of outcomes depends not only on the program design itself but also on the organizational and pedagogical context in which it is implemented^33,34,82^.

### Methodological considerations

Several methodological features strengthen the interpretation of our findings. The use of individualized thresholds for both heart rate and acceleration enabled relative classification of effort that accounted for differences in baseline fitness^44^. The unified three-zone intensity model was applied consistently across outcomes and aligned with health guidelines^45^, providing a coherent basis for interpretation. Including both perceptual (RPE) and affective (HHS) responses offered a multidimensional perspective on engagement, an aspect often overlooked in pediatric physical activity research. The structured design of the FIT FIRST FOR ALL sessions reflects widely recognized components of quality physical education, where developmentally appropriate design, instructional clarity, and consistent structure are emphasized as essential to pupil engagement and effective delivery^83^.

The study also has limitations. Although the intervention was implemented school-wide under normal school conditions, the acute analyses were conducted within one intervention school, which limits generalizability. The non-randomized cluster design increases the potential for confounding and restricts causal inference^36^. The acute subsample of 53 pupils allowed examination of overall response patterns, but its modest size, a male skew of 64%, and a distribution of baseline fitness skewed toward lower values reduced statistical power and increased the risk of type II errors. Distributional assumptions were addressed using non-parametric tests, yet subgroup findings should be interpreted cautiously as exploratory.

Peak accelerations were used to estimate external load, applying thresholds adapted from adult sprint-based frameworks. Individualized cut-points were used to contextualize estimates across pupils. For both heart rate and acceleration, limited pediatric-specific evidence exists to guide threshold selection. While wrist-worn accelerometer thresholds have been examined in children, less evidence is available for translational acceleration expressed in m·s^− 2^. The applied thresholds therefore provide relative indicators of external load rather than measures of maximal acceleration capacity. This limitation underscores the need for development and validation of pediatric-specific external load thresholds.

### Future directions

The acute response findings reinforce earlier results of health-related fitness improvements^26^ and physical well-being gains that were most evident among younger pupils and boys^27^. Taken together, these findings support the relevance of the FIT FIRST FOR ALL concept, which aligns with recognized components of quality physical education^6,7,23^. The sessions were structured, consistent, age-appropriate, and teacher-delivered, and the standardized format was designed to be simple, repeatable, and feasable within normal school conditions. These features may have supported fidelity and feasibility across schools and teachers^83,84^. Still, not all pupils may have benefited equally, and earlier analysis of health-related quality of life showed no significant effects beyond physical well-being^27^. This underscores the importance of further examining how pupils experience sessions and how implementation unfolds within school-wide settings. Future research should explore how to better support pupils who do not show clear gains despite participation and continue refining methodological tools used to evaluate session dynamics. Mixed-methods approaches, including qualitative exploration of classroom dynamics, perceived competence, teacher interactions, and delivery conditions may provide important insight into mechanisms of engagement and response. In addition, development and validation of pediatric-specific acceleration thresholds would strengthen the precision of external load classification and improve interpretation of intensity patterns in school-based physical activity research.

## Conclusion

The FIT FIRST FOR ALL program elicited acute responses characterized by health-promoting intensity and high enjoyment across pupils, including those with lower baseline fitness. Fitness improvements among lower-fit pupils were observed but were not statistically significant. These acute responses complement earlier findings of fitness and well-being benefits by indicating that pupils engaged with sufficient intensity and enjoyment to plausibly contribute to the improvements previously reported. Implemented school-wide and delivered by trained PE teachers, the structured, game-based design supported participation across age groups and session formats. Taken together, these features suggest simplicity, scalability, and alignment with quality physical education principles, making FIT FIRST FOR ALL a promising model for school-based physical activity promotion.

## Data Availability

The datasets generated and analyzed in this study are not publicly available due to privacy concerns in a small, closely connected population. Data are available from the corresponding author on reasonable request.
